# Supplementary material for: DMRforPairs: identifying Differentially Methylated Regions between unique samples using array based methylation profiles
Source: BMC Bioinformatics. 2014 May 15;15:141. doi: 10.1186/1471-2105-15-141 (PMC4046028; doi:10.1186/1471-2105-15-141)
Supplement: Additional file 2 — DMRforPairs output for the comparison of A431-MCF7 and NA17018-NA17105. Please start from the HTML files in each folder. Available via the BMC Bioinformatics website. [file 1471-2105-15-141-S2.zip › 1394847754114233_MOESM2_ESM/A431_MCF7/figures/13792.pdf]

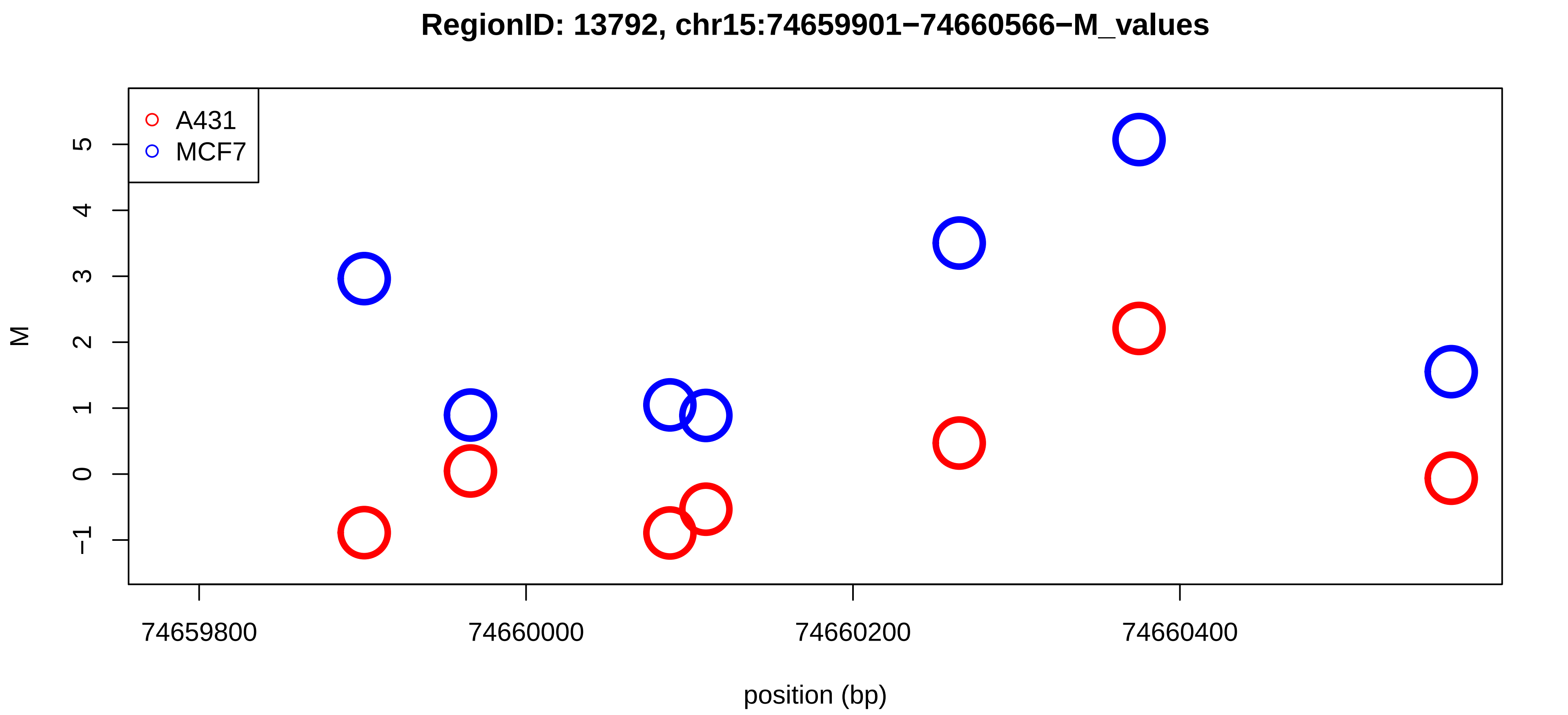
RegionID: 13792, chr15:74659901−74660566−M_values
A431
MCF7
M
−1
0
1
2
3
4
5
74659800
74660000
74660200
74660400
position (bp)

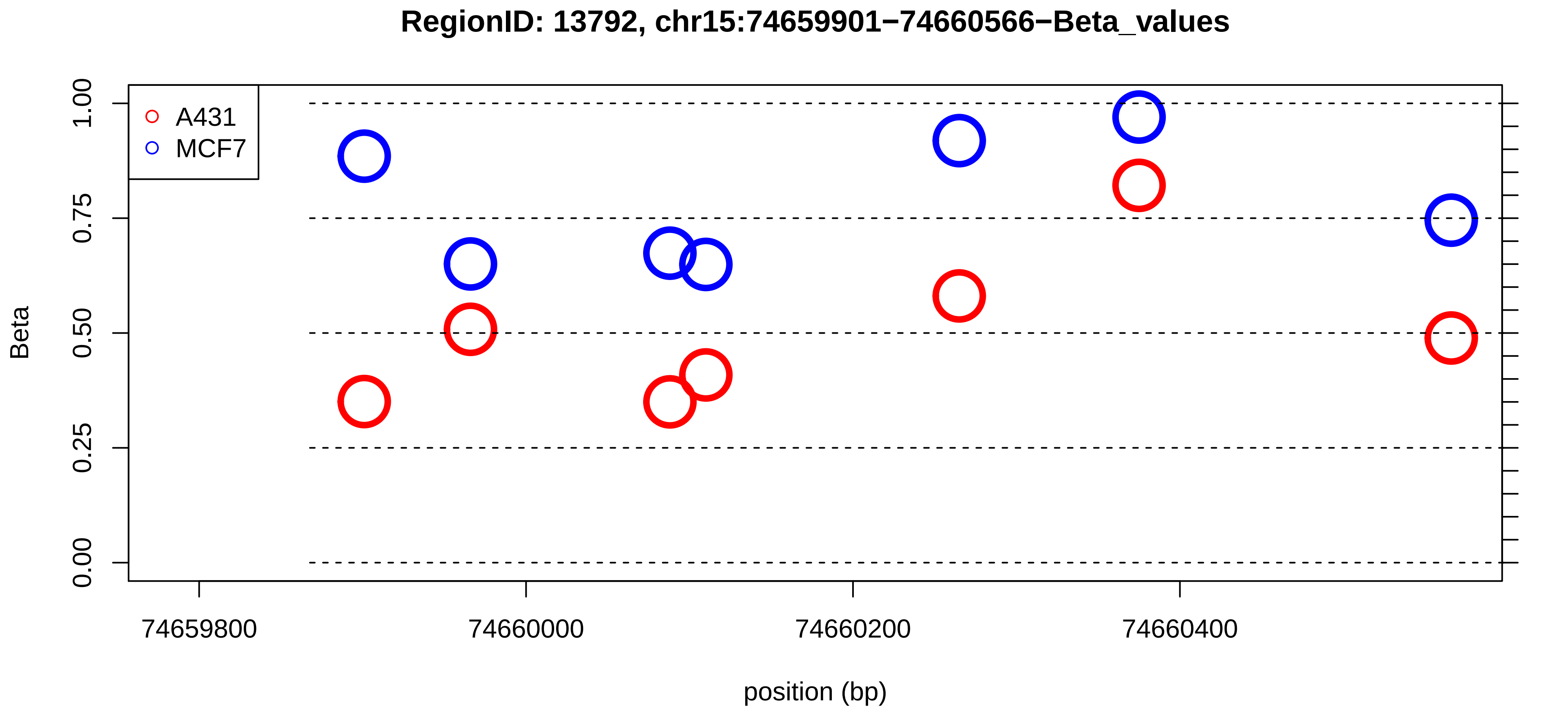
RegionID: 13792, chr15:74659901−74660566−Beta_values
A431
MCF7
Beta
0.00
0.25
0.50
0.75
1.00
74659800
74660000
74660200
74660400
position (bp)

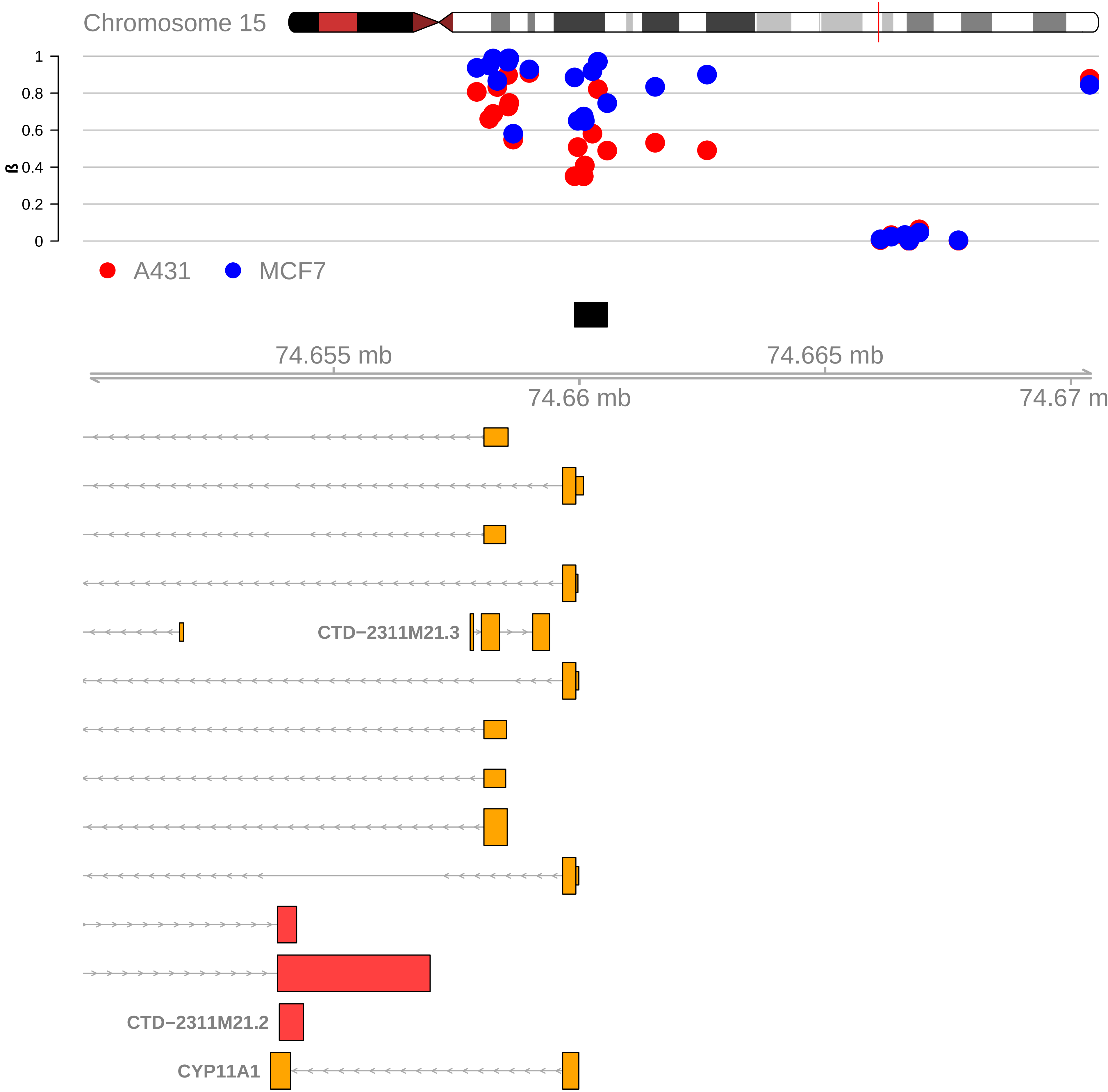
Chromosome 15
1
0.8
0.6
ß 0.4
0.2
0
A431
MCF7
74.655 mb
74.665 mb
74.66 mb
74.67 m
CTD−2311M21.3
CTD−2311M21.2
CYP11A1
